# Toward video-LLM driven workflow for behavioral segmentation and scoring in mice performing a skilled water-reaching task: an evaluation of recent LLM models

**DOI:** 10.1117/1.NPh.13.3.036601

**Published:** 2026-08-04

**Authors:** Tony Fong, Hao Hu, Haozong Zeng, Parnian Abbasi, Timothy H. Murphy

**Affiliations:** a University of British Columbia, Department of Psychiatry, Vancouver, British Columbia, Canada; b University of British Columbia, Djavad Mowafaghian Centre for Brain Health, Vancouver, British Columbia, Canada

**Keywords:** Large Language Model, video-Large Language Model, behavior classification, video analysis

## Abstract

Manual behavior scoring is labor-intensive and subjective. Video-capable large language models (LLMs) offer a transformative, scalable solution for accelerating and standardizing neuroscience workflows. We benchmarked state-of-the-art video LLMs (Gemini 2.5 Pro, Qwen3-VL, and VideoLLaMA3) for automated behavioral segmentation and scoring of mice performing a water-reaching task. Videos of mice performing water reaching were analyzed by the LLMs. Accuracy was compared across different models and against prompt adjustments within Gemini. To assess classification determinants, video fidelity was altered through pixel interpolation and key regions blurred (paws/snout-mouth). In addition, the models were asked to describe the mouse’s actions over time. Finally, an open-source rat lever-pressing dataset was utilized to validate behavioral segmentation under a few-shot learning framework, assessing the impact of visual examples on the identification of discrete action sequences. Gemini 2.5 Pro (0.74±0.12 accuracy) and Qwen3-VL-30B (0.67±0.13) exhibited the ability to classify trial outcomes. Reliable classification required a minimum pixel resolution of 0.28 mm per pixel and careful consideration of the model frame tokenization rate. Accuracy is significantly reduced upon obscuring the snout-mouth area. In 549/1058 of videos, Gemini 2.5 Pro also provided completely accurate frame-to-frame behavior segmentations. The inclusion of visual examples improved model detection of user-defined behaviors. Video-LLMs offer potential to accelerate neuroscience by providing scalable, objective quantification of goal-directed behaviors. By producing temporal annotations, Gemini enables fast first-pass labeling that markedly streamlines manual dataset curation.

## 1 Introduction

In neuroscience, behavior remains the primary outcome of interest across many diverse subfields. As such, quantitative and unbiased assessments of animal behavior are critical, yet traditional scoring methods are not only laborious, but also prone to human errors and biased by subjective interpretation, even with corrective efforts.^1^^–^^3^ Over the past decade, computer vision methods—most notably convolutional neural network (CNN)-based frameworks such as DeepLabCut, SLEAP, and Lighting Pose—have revolutionized behavioral studies by enabling markerless pose estimation in 2D and 3D.^4^^–^^9^ When combined with clustering algorithms that group keypoints into behaviorally meaningful states, these methods have enabled supervised and unsupervised classification of specific behaviors.^10^^–^^15^ These approaches represent an important step toward standardization and reproducibility in behavioral neuroscience. However, pose-based clustering alone remains insufficient for understanding how animals interact with the environment—particularly in trial-based tasks where outcomes (e.g., hits versus misses) are critical. Tools such as capacitive sensors,^16^^,^^17^ IR beam breaks,^18^ and motor encoders^19^ are well suited for low-dimensional tasks (e.g., lever pressing) but introduce significant constraints in less restrictive paradigms. Rotary encoders require a physical manipulandum, which can alter natural reach kinematics and may obstruct the visual field. Similarly, lick sensors and infrared beam-breaks primarily measure physical contact or movement rather than task success; these binary signals provide only an indirect proxy that cannot reliably report functional trial outcomes. One such task is the skilled forelimb reach-to-grasp and retrieval assay; in this context, true task success cannot be reliably inferred from simple binary signals (water spout contact), which fail to capture the full, integrated sequence of reaching, retrieval, and consumption.^20^^–^^23^ For example, a lick sensor may register a "false positive" if the animal contacts the spout without successfully retrieving the water, or a "false negative" if the animal retrieves and consumes the water directly from its paw without triggering the sensor. Consequently, sensor-based approaches may not be suitable for all behavioral paradigms. At present, there are no robust out-of-the-box video-based approaches that do not require fine-tuning that can distinguish successful from failed reaching attempts. By contrast, video-based LLM scoring evaluates the full, goal-directed behavioral sequence, enabling accurate outcome classification without imposing physical constraints or relying on indirect, approximated signals.

In recent years, the rapid evolution of large multimodal models (LMMs) has opened new frontiers in video understanding.^24^ Many LLM-based models, such as VideoPrism^25^ from Google DeepMind and MouseGPT,^26^ can achieve human-level performance in pose estimation and self-directed behavior classification from raw videos alone, reaching parity with expert scorers on benchmark datasets of mouse videos. These advances suggest that general-purpose video LLMs trained on large and diverse datasets may go beyond recognizing self-directed behaviors to classifying environmental interactions and their resulting outcomes. However, these approaches typically require extensive fine-tuning of downstream video encoders or task-specific classifiers to generalize across different views or behavioral paradigms. Most recently, the release of Gemini 2.5 Pro^27^ and Qwen3-VL^28^^,^^29^ models surpasses prior models across a range of video understanding benchmarks and raises the possibility that video LLMs could generalize to mouse behavior scoring without additional training.

Here, we introduce a workflow that leverages video LLMs to directly segment and score rodent reach-to-grasp behaviors from single-view videos. This approach lays the foundation for providing an efficient and scalable alternative to traditional pipelines that rely on pose estimation, feature engineering, and clustering. Using this framework, we benchmark several recent video LLMs on head-fixed mice performing a water-reaching task. Our results suggest that video LLMs may streamline behavior analysis, reduce hardware and annotation overhead, and offer fully generalizable scoring capabilities for behavioral neuroscience.

## 2 Methods

### 2.1 Animals and Surgery

Male C57BL/6 mice, 5 to 6 months of age and of varied genotypes, were used in this study. Animals were maintained under a standard 12/12  h light/dark cycle (lights on at 7:00 A.M.). Mice were implanted with a chronic transcranial window and a head-fixation bar as previously described.^30^ All experimental procedures were approved by the University of British Columbia Animal Care Committee and were conducted in accordance with national guidelines.

### 2.2 Water Reaching Training and Testing

**Fig. 1:**
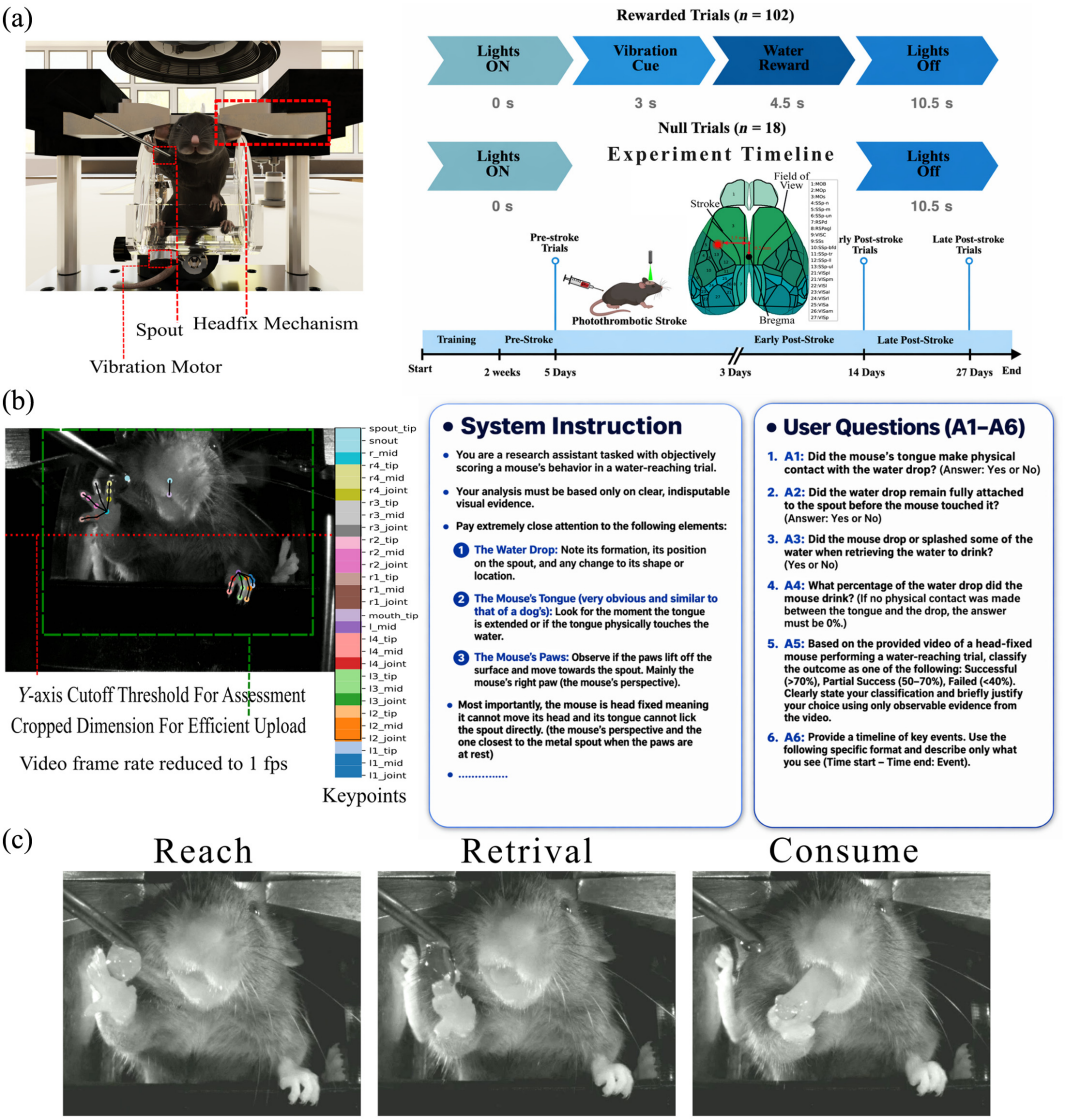
Experimental setup and LLM input. (a) Mouse water-reaching task and experimental timeline. Mice were headfixed in a custom setup in a standing position. A vibration motor was situated on the cup. An animal underwent 102 rewarded trials and 18 nonrewarded trials in each session. A vibration reward was delivered 3 s after a trial start followed by a water reward 1.5 s later. Six seconds after the reward the trial ends. Results of the trial are scored on −1.5 and 4.5 s after the reward is delivered. Mice trained for a minimum of 2 weeks prior to testing and received stroke induction (1.5; 0.5) mm from bregma. (b) Video data configuration with system instructions and applied prompts. The original video was cropped for efficient transfer to Gemini. Only trials where the mouse’s right paw, tracked by DeepLabCut, rises above a certain threshold are scored. (c) Example phases of the reach in a single trial. All video LLMs received system instruction and user questions with the video input (Video 1, MP4, 484 KB [URL: https://doi.org/10.1117/1.NPh.13.3.036601.s1]).

Following post-surgical recovery, mice were water-restricted and trained on a custom head-fixation platform [Fig. 1(a)]. Each session consisted of 120 trials, including 102 potentially rewarded trials (85%) and 18 null trials (15%). Approximately 10  μl of water reward was delivered through a spout positioned 6 mm to the right of the animal’s snout (vertical offset±2  mm) controlled by a raspberry pi 4B GPIO using custom scripts. Successful trials were defined as trials in which the mouse successfully reached for, retrieved, and consumed the water drop, as illustrated in Fig. 1(c). An illustrative example is provided in Video 1. Mice underwent a minimum of 3 weeks of training prior to testing. After 7 days of baseline testing, a photothrombotic stroke was induced, followed by a 3-day recovery period. Testing then resumed once daily for an additional 24 days. Behavior capture was conducted using a global shutter raspberry pi camera (IMX296 monochrome Innomaker) recorded at a resolution of 1200×800 at 54 fps with 5 ms exposure under IR light to capture without motion blur.

### 2.3 Photothrombotic Stroke

Focal ischemia was induced by photothrombotic occlusion at a target region between the sensory and motor cortex [stereotaxic coordinates: 1.5 mm lateral, 0.5 mm anterior to bregma; Fig. 1(a)] as previously described.^31^ Mice received an intraperitoneal injection of the photosensitive dye Rose Bengal (RB; 0.1  ml/10  g body weight; R3877-5G, Sigma-Aldrich, St. Louis, Missouri, United States). Two minutes later, a 40 mW diode-pumped solid-state 532 nm laser, attenuated to 17 mW with a polarizer, was applied to the target region to induce focal ischemia. The laser beam diameter was 0.9 mm at full width at half maximum. Prior work has shown that this procedure produces tissue damage restricted to the irradiated area.

### 2.4 Videos Processing Pre-LLM Analysis

As shown in Figs. 1(b) and 1(c), only the 1.5 s period before and 4.5 s after reward delivery from each video was selected for scoring. All digits from both paws, snout and mouth tip, were tracked using DeepLabCut.^7^ Only videos in which the center of the reaching paw (average of all digits for the right paw) moved above a manually defined y-axis threshold were included in the analysis. To optimize upload speed, the videos were cropped to a resolution of 720×740  pixels.

### 2.5 Video Manipulations

To test the effect of temporal resolution needed, videos were also re-encoded frame by frame using opencv-python (v4.8.0.76) to specific frame rates of 1, 2, 6, 13, 27, and 54 fps. Gemini would only sample/tokenize one frame per second of video playtime by default, which translates to the number of frames it samples as 1/fps, i.e., the slower the frame rate, the more frames Gemini would sample. However, to ensure all frames would be tokenized by the tested video LLM (Gemini, Qwen, and VideoLLaMA), all analysis was conducted on videos set to 1 fps unless otherwise indicated. For testing changes in video resolution, each clip was first down sampled and then up sampled back to the original dimensions prior to model input to maintain a consistent total token count. This procedure reduces the effective video resolution because fine-grained spatial details lost during down sampling cannot be recovered through up sampling, resulting in a visually smooth but information-reduced video that preserves size but not detail. To evaluate the importance of localized visual cues, the centers of the right and left paws were blurred using a Gaussian blur (σ=0, radius=140  pixels, kernel size=51). The center of the mouse face (estimated as the average pixel position between the snout and mouth tip) was blurred using a Gaussian blur (σ=0, radius=180  pixels, kernel size=51).

### 2.6 Video LLM Analysis

To comprehensively evaluate zero-shot behavioral classification, we selected models representing different scales and architectures within the current video-LLM landscape. Gemini 2.5 Pro was selected as a representative proprietary, exceptionally large-parameter model known for its robust spatial-temporal reasoning and massive context window. For open-source comparisons, we utilized Qwen3-VL (at both 30B and 8B parameter scales), which currently represents the state-of-the-art in open-source vision-language understanding with strong capabilities in fine-grained visual grounding. Finally, VideoLLaMA3 (7B) was included as a representative baseline for smaller, accessible open-source multimodal architectures. Comparing these models allows us to evaluate the trade-offs between model scale, proprietary accessibility, and classification accuracy.

**Fig. 2:**
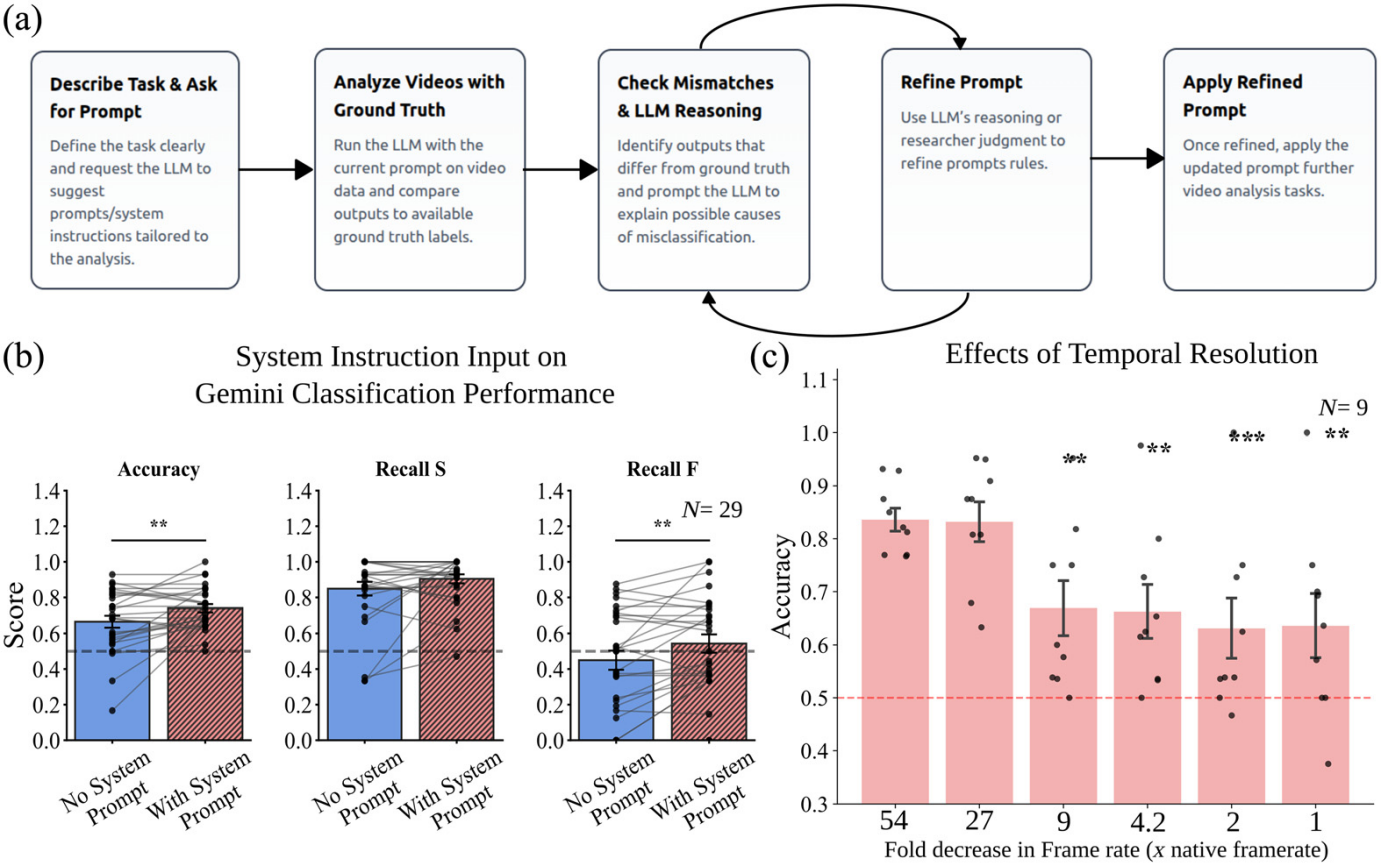
LLM workflow and parameters testing. (a) LLM prompt generation and refinement for behavioral video analysis. Only the system instructions of the prompt were continuously refined during each interaction. For simplicity, system instructions were refined in Google AI Studio. (b) Improved accuracy of LLM with system instructions/prompt refinement and increased temporal.* The same sessions (with 6 to 88 videos each) are analyzed with or without system instructions added. (c) Videos were set to a given apparent frame rate (fold decrease compared with native framerate of 54 fps) in opencv prior to delivery to Gemini. The lower the frame rate, the more frames the LLM would sample. The red dashed line represents the performance of a random classifier. *p<0.05, **p<0.01, and ***p<0.001. Each point represents a session composed of 6 to 88 trials.

Prompts used in the analysis were generated by the LLM itself using descriptive guidance by one of the authors. Figure 2(a) illustrates the overall process for prompt generation, detailing both the system instructions and user questions. Only the system instructions were modified in each iteration, whereas user questions were adjusted solely for consistency of flow. System instructions were refined in Google AI Studio.^32^ The full system instructions applied can be found in Fig. S1 in the Supplementary Material. The model and human expert results classified trials as success (visually consumed >70% of the water reward), partial success (50 to 70% water consumption), and failure (<40% of water consumed), as defined in the prompt [Fig. 2(a)]. For analysis, partial and full successes (for water drop consumption per trial) were combined into a single success category to simplify scoring and comparisons.

For Gemini 2.5 Pro, video analysis (i.e., uploading the system instruction prompt and videos) was performed using the Google Gen AI SDK (https://github.com/googleapis/python-genai). Detailed documentation can be found in Ref. 33. A custom scheme was also created and uploaded for structured outputs from Gemini. All other open-source video LLM models were run in Python using the transformers (v4.57.1) package on a workstation equipped with a GPU featuring 96 GB of video memory (RTX-PRO-6000). Weights for Qwen3-VL-30B, Qwen3-VL-8B, and VideoLLaMa3 were retrieved from Hugging Face with the following links Qwen/Qwen3-VL-30B-A3B-Instruct, Qwen/Qwen3-VL-30B-A3B-Instruct-FP8, Qwen/Qwen3-VL-8B-Instruct, and DAMO-NLP-SG/VideoLLaMA3-7B, respectively. In the Qwen and VideoLLaMa models, system instructions were input as past chat history. Detailed instructions for employing these open source models can be found at their respective GitHub page: Qwen (https://github.com/QwenLM/Qwen3-VL) and VideoLLaMA (https://github.com/DAMO-NLP-SG/VideoLLaMA3) both Alibaba associated repository https://github.com/QwenLM and https://github.com/DAMO-NLP-SG, respectively. All videos were analyzed with the temperature set to 0 (or 0.1 if 0 is not a valid option) to maintain consistency and top-p to 0.95 to minimize hallucinated outputs. Specific system instruction can be found in Fig. S1 in the Supplementary Material.

### 2.7 Step-by-Step Analysis Protocol

To facilitate the replication of our video-LLM pipeline by other laboratories, the exact workflow is summarized in the following five steps:

1. Video pre-processing and bout extraction: Raw behavioral videos are temporally cropped (1.5 s prior to and 4.5 s following reward delivery) based on reaching bouts identified via DeepLabCut. Videos are then spatially cropped to 720×740  pixels to optimize data handling.
2. Frame rate optimization: Videos are re-encoded to 1 frame per second (fps) using opencv-python. Models like Gemini natively sample 1 frame per second of video playtime; re-encoding ensures every visual frame is tokenized.
3. Prompt engineering and system instructions: Task-specific grading criteria (success, partial success, failure) are drafted and iteratively refined using a subset of varied videos via a GUI interface (e.g., Google AI Studio) to ensure the model attends to the correct salient features.
4. Model inference setup: For proprietary models (Gemini 2.5 Pro), videos and instructions are submitted via the official API using a strict structured JSON output schema. For open-source models (Qwen-3-VL, VideoLLaMA3), weights are downloaded via Hugging Face and run locally using the transformers library.
5. Execution and output parsing: Across all models, generation temperature is set to near-zero (0 or 0.1) for deterministic reproducibility, and top-p is set to 0.95. Structured text outputs are subsequently parsed via Python scripts for statistical comparison against human annotations.

### 2.8 Gemini 2.5 Pro Water Drop Quantification

To assess Gemini 2.5 Pro’s quantification ability, real still images of water drops of varied volumes (20, 40, 80, and 160  μl) on a parafilm sheet were presented to the model. The model was instructed to detect both the number of water drops and their relative sizes, giving more weight to the smallest discernible drops, to evaluate its capacity for recognizing relative amounts.

To further test the model’s evaluation of temporal changes in quantity, videos of a single 80  μl water drop undergoing volumetric manipulation were presented (using a pipette). In these videos (recorded at 30 fps), the drop’s volume was changed by +25%, 0, −25%, −50%, −75%, and −100% of its initial volume of 80  μl. The model was then queried on the estimated percentage of water removed from the original 80  μl volume.

### 2.9 Rat Lever Pressing Analysis

Open-source videos of rats executing a lever-pressing task were extracted from the Supplementary Materials (Videos 2 and 3) of Kawai et al. (2015)^34^ and assessed by video LLM. The primary behaviors targeted for segmentation in the open-source dataset were lever presses and reward-drinking events. A total of 20 videos were extracted and manually annotated for the events. Models were explicitly tasked with predicting the timing and duration of discrete action sequences. Performance was compared between zero-shot prompting (providing only system instructions) and a few-shot learning framework with context information. In the few-shot condition, the model’s chat history was augmented with example context images explicitly identifying snout locations and corresponding target actions to establish visual anchors for the target behaviors.

#### 2.9.1 Statistical analysis

Data are all presented as mean ± std. Statistical significance was determined using a post hoc two-way ANOVA followed by paired Student’s t tests (with Tukey’s correction) as appropriate in Python. Single descriptor events are modeled as Bernoulli/ Binomial processes to calculate mean and 99% confidence intervals. Cohen’s kappa (κ) was calculated as κ = (observed agreement-expected agreement by chance) / (1-expected agreement by chance), measuring inter-rater reliability beyond chance agreement. Values were interpreted using Landis and Koch (1977) benchmarks: 0.41 to 0.60 fair agreement, 0.61 to 0.80 as substantial agreement, and 0.81 to 1.00 as almost perfect agreement. Prior to calculating accuracy and F1-scores, data were balanced by randomly down sampling to the minority class size within each session to ensure equal representations when benchmarking. For example, if a session with only 20 failed trials and 50 successful trials, the dataset is balanced by selecting a random subsample of 20 successful trials (and all failed trials) for the analysis. All performance metrics were calculated within each session, containing 6 to 88 trials. The level of significance is denoted on the figures as follows: *p<0.05, **p<0.01, and ***p<0.001.

## 3 Results

Mice were trained for a minimum of 2 weeks on a head-fixed water-reaching task. During training, the water spout was positioned ∼6  mm lateral to the right, 2 mm ventral, and 5 mm anterior relative to the mouse’s snout. Each rewarded trial began with a vibration cue, followed by water delivery 1.5 s later. Videos were scored from 1.5 s before to 4.5 s after reward delivery. Nonrewarded trials were not scored, as illustrated in Fig. 1(a). To optimize performance, we implemented an iterative workflow for prompt engineering [Fig. 2(a)]. Prompts provided to Gemini consisted of two components: system instructions and user queries. The system instructions served as predefined guidelines that directed the video LLM’s reasoning process and shaped its output. We iteratively tested Gemini’s responses on 10 trial videos with known ground truth (human) labels, identified incorrect outputs, and refined the system instructions accordingly. Each iteration incorporated corrections and model feedback until the generated outputs aligned with the ground truth. A paired t-test revealed that the presence of system instructions affected the accuracy (0.07 increase; p<0.01), F1-scores for failure classification (0.09 increase; p<0.01), but not success classification (0.06 increase; p=0.1) of Gemini [Fig. 2(b)].

Next, we investigated the effect of temporal resolution on Gemini outputs. By default, Gemini only samples or tokenizes one frame each second (of the video playtime). Therefore, we adjusted the video input (original recording 54 fps) to 1, 2, 6, 13, 27, and 54 fps, which corresponds to a 54, 27, 9, 4.2, 2, and 1 time slowing of frame rates with as shown in Fig. 2(c). Using a paired t-test, frames rates of 6 (p<0.01), 13 (p<0.01), 27 (p<0.001), and 54 (p<0.01) decreased the accuracy of Gemini classification [Fig. 2(c)]. Although at 2 fps (p=0.9) did not significantly alter mean accuracy of the model, it increased the variability, i.e., standard deviation, from 0.07 (1 fps) to 0.11.

**Fig. 3:**
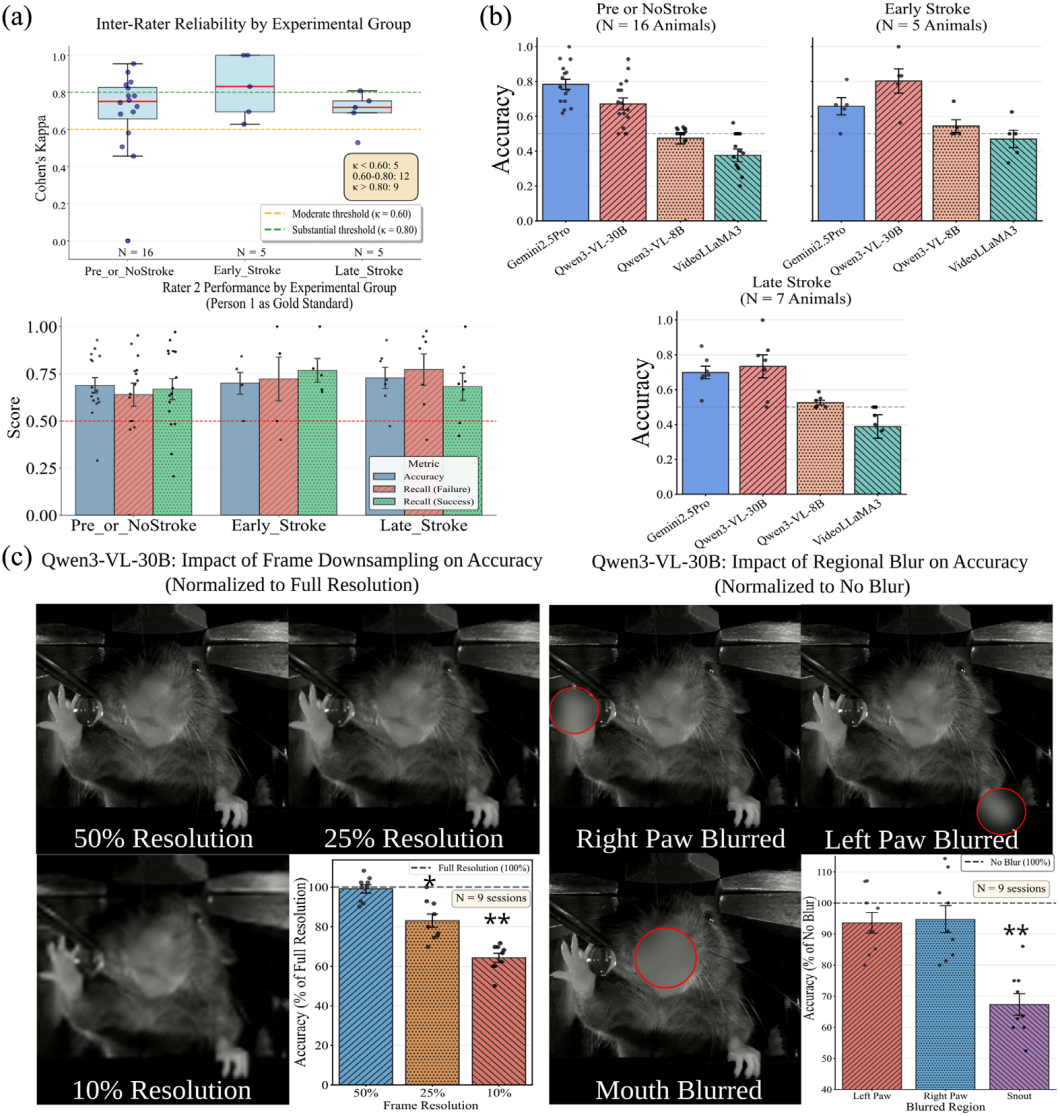
Model performance on water-reaching task. (a) Inter-rater reliability, as measured by Cohen’s kappa, between two expert human scorers across different experimental groups: Pre-or-no-stroke, early stroke, and late stroke. The dashed lines indicate moderate (κ=0.60) and substantial (κ=0.80) agreement thresholds and number of sessions that passed the corresponding threshold. (b) Accuracy of each tested model in classifying water-reaching trials across experimental groups. Data are calculated per session (N), which consists of multiple trial videos. (c) The effect of video resolution and part specific blurring on model performance. The left panel shows example frames at 50%, 25%, and 10% of the original resolution. The bar chart on the right shows the corresponding drop in classification accuracy compared with the original, un-blurred video (green dashed line). Each point represents a session of 6 to 88 trials; the ground truth is expert hand-scored results. Data are mean ± SEM. (b) Comparison of Gemini and human accuracy after corrections. Data are mean ± std. (c) The blurred region is enclosed by a red circle. *p<0.05, **p<0.01, and ***p<0.001. Each point represents a session composed of 6 to 88 trials.

The inter-rater analysis demonstrated that trained (and blinded to outcome) human scorers exhibited similar levels of inter-rater agreement, as measured by Cohen’s kappa, when evaluating mice in the pre-stroke or no-stroke condition (0.693±0.057), during the early-stroke phase (0.831±0.076), and in the late-stroke phase (0.7±0.047) [Fig. 3(a)]. However, only nine sessions achieved kappa values above 0.80 (substantial agreement), whereas five sessions fell below the moderate agreement threshold of 0.60. These observations indicate the presence of scorer bias and variability that can influence outcome assessments. When treating rater 1 (who had the most experience) as the gold standard, rater 2’s performance remained robust, achieving kappa values greater than 0.80 across all conditions, as shown in Fig. 3(a). Given these findings on agreement and accuracy, all the following results are compared to rater 1 as the gold standard. Kappa values were also calculated for differences between Gemini and human rater 1. Although a lower average Kappa value of 0.43±0.24 was found between Gemini and rater 1, this Kappa value indicates that there is still an overall fair agreement. Notably, 6/16 (pre-stroke or no-stroke condition animals) were in strong agreement (Fig. S2 in the Supplementary Material).

Performance of the four video LLM models on the accuracy (versus a rater 1 as the gold standard) of scoring water-reaching success of mice performing across pre-stroke, early-stroke, and late-stroke animal groups is shown in Fig. 3(b). A two-way ANOVA (model × group) revealed a highly significant overall difference in classification accuracy among the models (F=1.795627, p<0.001). Post-hoc analysis with Tukey’s HSD correction identified: Qwen3-VL-30B (0.71±0.15) and Gemini 2.5 Pro (0.67±0.18) demonstrated higher (p<0.001) accuracy compared with Qwen-3-VL-8B (0.50±0.11) and VideoLLaMA3 (0.40±0.15). Further post-hoc analysis revealed that Qwen-3VL-30B had a higher accuracy compared with Gemini 2.5 Pro in evaluating success and failure in mice during early-stroke phases. Although some differences between the mean accuracy measures of Gemini 2.5 Pro and Qwen3-VL-30B were observed across different treatment groups, no statistical significance was found (model × group interaction effect: p=0.12). Taken together, these statistical results identify Qwen-3VL-30B and Gemini 2.5 Pro as the only accurate models tested for the automated analysis of this behavioral task. Further looking into the specific classification, F1-scores for failure and success classification seem to be more imbalanced in Qwen3-VL-8B and VideoLLaMA3 than Qwen3-VL-30B and Gemini 2.5 Pro (Fig. S3 in the Supplementary Material) and may explain the differences observed in accuracy measures. Considering cost of analysis (∼0.5 USD per video) and given similar performance, further analyses were then conducted with the Qwen-3VL-30B model.

Next, the importance of visual information for trial classification was evaluated in the Qwen-3VL-30B model. Nine sessions with the highest baseline accuracy were selected, and their trial video clips were systematically altered in resolution or feature blurring as described below. To reduce effective resolution while controlling total token input to the model, videos were first down sampled and then resized to their original resolution through bilinear interpolation. Reducing the resolution to 25% (mean accuracy=83.0%, p=0.011) and 10% (accuracy mean=64.3%, p=0.004) of the original resulted in a significant, resolution-dependent decrease in model accuracy [Fig. 3(c) left], as determined by the Wilcoxon signed-rank test. Using these blurring procedures, we estimated that pixel sizes >0.28 mm led to decreases in accuracy.

The effect of selectively blurring key body regions was also examined on accuracy. Blurring the right paw (mean=94.8%, p=0.20) or left paw (mean=93.6%, p=0.21) did not produce statistically significant changes in accuracy. By contrast, blurring the snout region significantly impaired model accuracy (mean=67.4%, p=0.039), indicating that orofacial cues are critical for discriminating successful versus failed trials. The results are illustrated in Fig. 3(c) right. However, when the input was further constrained to only the isolated orofacial region, model performance collapsed, with all trials classified as failures (Fig. S4 in Supplementary Material). This indicates that although orofacial cues are necessary, they are not sufficient in isolation.

**Fig. 4:**
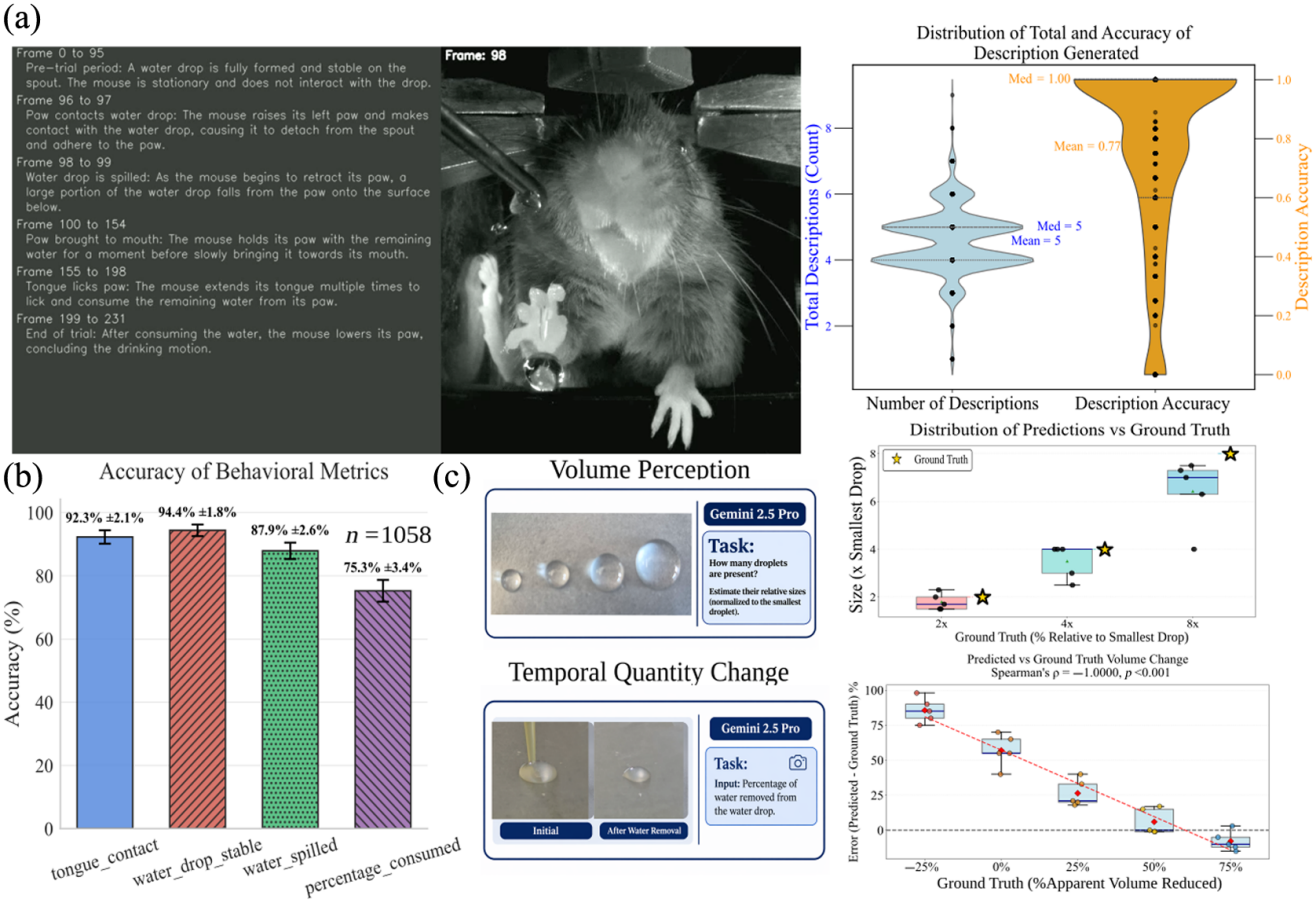
Gemini 2.5 pro analysis performance on head-fixed mouse water-reaching task and water drop quantification. (a) Example of behavior segmentation by Gemini 2.5 Pro. Right: Comparison of classification accuracy between human annotators and Gemini after corrections. Each point represents an individual session; summary values are shown as mean ± SEM. (b) Gemini performance in video segmentation (frame classification by action/content). Data are mean±99% CI. The red line shows random (chance-level) accuracy. (c) Evaluation of Gemini to quantitatively detect water drop sizes in still images and volume changes in videos. Images of water drops were presented and the model estimated size compared with the smallest drop (top graph). Videos showing varying percentages of volume removal from a 80  μl water drop were presented to evaluate the model’s ability to track changes in water drop size (bottom graph). In this case, large volume reductions simulating consumption of water by the mouse were reported with lower error.

Given that the capabilities of video LLMs can extend well beyond simple classification,^35^ we examined whether they can provide rich, frame-level descriptors of behavior without fine-tuning. Due to the accuracy being highest in no-stroke or pre-stroke animals for Gemini 2.5 Pro, we further analyzed additional outputs from the prompt for all trial videos (n=1058) in these animals. First, Gemini was asked to provide answers for the following descriptors:^1^ tongue_contact (whether the tongue touched the water reward),^2^ water_drop_stable (whether the water drop remained stable after delivery),^3^ water_spilled (whether water spilled from the paw during the task),^4^ percentage_consumed (visual estimate of how much of the water drop was consumed), and^5^ timeline of events (frame-by-frame event classification and descriptions). These outputs were manually evaluated and curated by an expert (with access to all outputs of the video from Gemini 2.5 Pro) and deemed accurate only when the scorer agreed with the model’s result on the video evaluated. An illustration of these outputs is shown in Figs. 4(a) and 4(b).

Importantly, Gemini also provides temporal localization of events with per second resolution. As stated, before the temporal resolution of behavior events can be further enhanced by manipulating the sampling rate in frames, the default for Gemini is to sample at 1 Hz by skipping frames. The model can be used in a higher temporal resolution mode by re-sampling the time base: for example, if frames were collected at 54 Hz for 1 s, the time per frame can be reset to create 54 s of 1 Hz data so that all frames will be used. As illustrated in Fig. 4(a), Gemini can detect brief events, such as water spillage lasting only 1 to 2 frames. Also shown in Fig. 4(a), Gemini generates on average five descriptions per video (median=5). A deeper analysis revealed that the average per-video accuracy or in agreement with the evaluator of these descriptions (correct descriptions / total descriptions) was 0.77, with a median of 1.0; notably, 549 of 1058 videos contained entirely correct descriptions. All 1058 videos are available at the current link (^36^). For the descriptions tongue_contact, water_drop_stable, and water_spilled, Gemini achieved high accuracies of 92.3±2.1%, 94.4±1.8%, and 87.9±2.6%, respectively, indicating these events were detected reliably and consistently. By contrast, percentage_consumed showed lower agreement with expert scoring likely because Gemini either may not perceive quantitative amounts or cannot relate visual changes to quantitative measurements. This suggests that Gemini captures key instructed behavioral events robustly but has more difficulty with fine-grained quantitative judgments.

To evaluate the Gemini ‘s quantitative abilities, it was presented with images of four water drops of increasing volumes (20, 40, 80, and 160  μl) and tasked with determining their sizes relative to the smallest drop [Fig. 4(c)]. Although the model was able to correctly detect the number of drops in all test cases (N=5), it was not precise in measuring the exact relative volumes, showing a tendency to underestimate. However, the model demonstrated a clear ability to discern relative sizes, as its predictions showed a significant positive linear correlation with the actual volume (Pearson’s r=0.57, p=0.03). When presented with videos depicting volume reduction in an 80  μl water drop, the model struggled to distinguish small percentage changes. Interestingly, although Gemini struggled to predict small additions or removals (±25% volume), its error rate for removals decreased linearly (Pearson’s r=−0.94, p<0.01) as the amount of water removed increased indicating lower error near the 70% consumption threshold used to score videos.

**Fig. 5:**
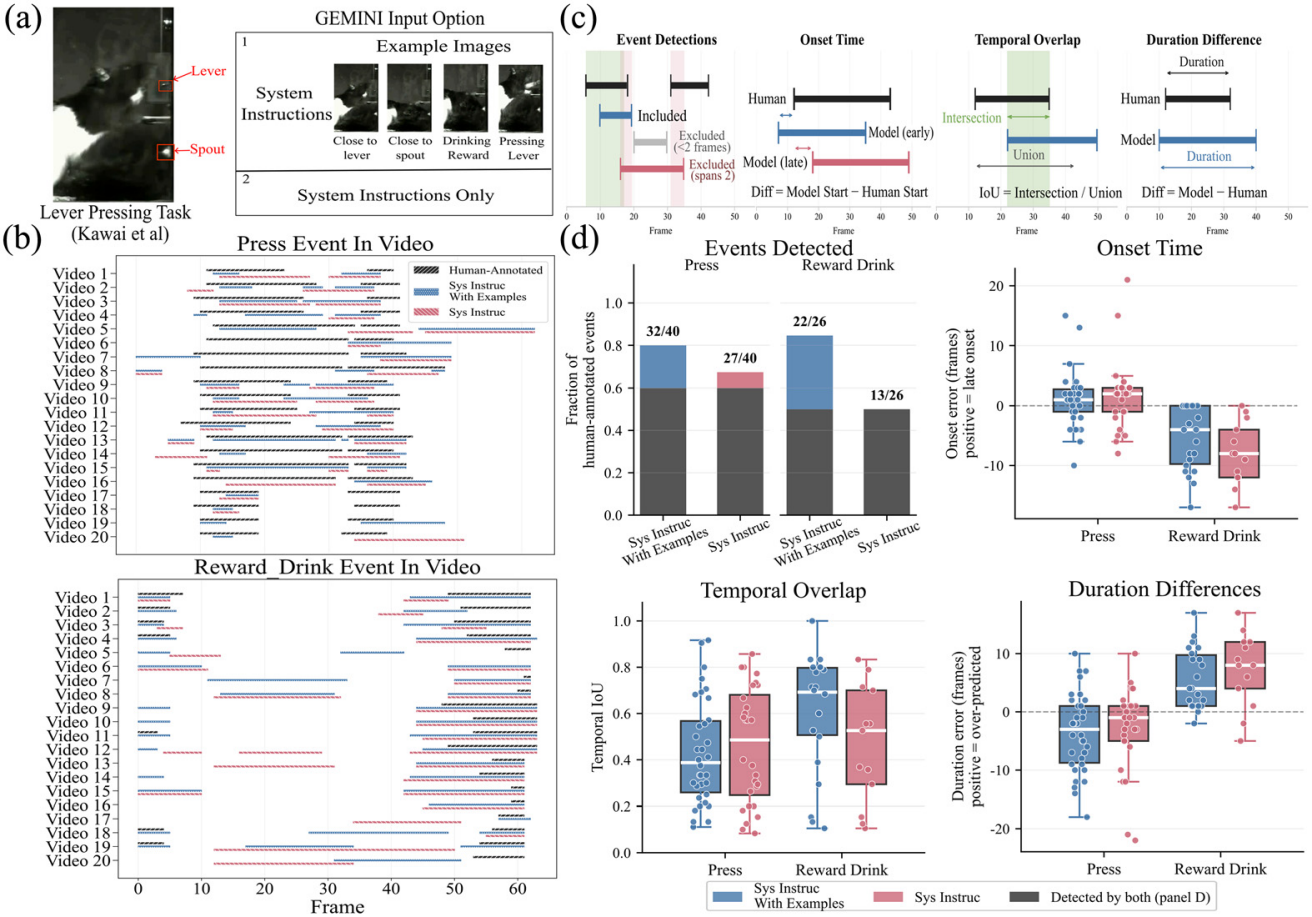
Gemini evaluation on a rat lever-pressing task (Kawai et al., 2015). (a) Left: Behavioral chamber showing the lever and reward spout (two presses required for a reward). Right: Gemini input conditions comparing system instructions with example images against system instructions only. (b) Raster plots of predicted (Gemini under two modes) versus ground-truth (black) for all events for “press” and “reward drink” across 20 videos. (c) Schematics defining evaluation metrics: Event detections (inclusion requires at least a 2-frame overlap with a single human event; predictions with <2 frames overlap or spanning multiple events are excluded), onset time (early/late prediction in frames), temporal overlap (intersection over union, IoU), and duration difference (under/over-prediction in frames). (d) Temporal localization performance comparing the example-based (blue) and text-only (pink) models for events that pass criterion from panel (c). Events detected displays the fraction of successful human-annotated matches. The remaining panels quantify the accuracy of these matched detections via onset time error, temporal overlap (IoU), and duration differences. No statistic was found between the models (two-sided Welch independent-samples t-test, p>0.05). Individual scatter points represent individual events detected.

To further evaluate the capacity for automated behavioral segmentation, Gemini 2.5 Pro was tasked with identifying the timing and duration of lever press and reward-drinking events from an open-source dataset (Kawai et al., 2015). Performance was compared between two prompting conditions: a zero-shot approach providing only system instructions, and a few-shot approach augmented with example images explicitly denoting snout location and target actions [Fig. 5(a)]. Qualitative assessment of the event raster plots [Fig. 5(b)] indicates that model predictions tracked human-annotated ground truths across the 20 video samples. To quantitatively measure detection quality, event detection criteria, event onset, temporal overlap (IoU), and duration differences relative to human annotations were evaluated, as depicted in Fig. 5(c). To determine whether these detection capabilities reflected random temporal alignment, we compared LLM-predicted event onsets with a random prediction model. LLM detections were more closely aligned with human annotations than expected by chance, supporting meaningful temporal correspondence between model predictions and expert annotations (Fig. S5 in the Supplementary Material).

Notably, the inclusion of visual context examples in the prompt history improved the overall event detection rate [Fig. 5(d), top left]. For discrete lever press events, the detection rate increased from 27/40 (67.5%) under standard instructions to 32/40 (80%) with examples. This enhancement was particularly pronounced for the subtler motor kinematics of reward-drinking events, where successful detection increased from 13/26 (50%) to 22/26 (84.6%). Despite these behavior-specific shifts, the overall temporal overlap (IoU) for successfully detected events remained comparable across both target actions (two-sided Welch independent-samples t-test, p>0.05), supporting the model’s viability for temporal behavioral localization. However, it is possible that this observed consistency is due to the analysis being underpowered from a small sample size. [Fig. 5(d), bottom left].

## 4 Discussion and Conclusion

Interestingly, trained human scorers can reliably classify trial outcomes without 3D reconstruction or multiview recordings as we have shown; instead, they rely on a small set of salient visual cues that are often stable across camera setups. This observation suggests that accurate behavior scoring depends less on full kinematic reconstruction and more on extracting task-specific, visually unmistakable events. In this context, the current study provides a comprehensive evaluation of three prominent large language models for automated classification of trial outcomes in a head-fixed mouse water-reaching task. By directly comparing their performance to expert human annotations, we assess whether video-language models can capture the essential visual information needed for determining task success, despite the absence of explicit pose tracking or depth information. The results demonstrated that Qwen-3-VL-30B and Gemini 2.5 Pro were the strongest performers and suggest that prompt engineering techniques can influence the quality of outputs. Gemini 2.5 Pro achieved the highest overall accuracy, but this performance was not statistically superior to Qwen3-VL-30B when all experimental groups were combined. However, notable differences emerged in stroke-specific performance, as discussed below. Notably, within the Gemini 2.5 Pro results, accuracy in the no/pre-stroke condition was trended higher than in the early-stroke and later-stroke condition. Classification performance—F1-score—for the success category remained superior to that for failure across all sessions and conditions for the Gemini 2.5 Pro model. This likely reflects the fact that successful water consumption presents clear, consistent, and robust visual cues (e.g., paw movement, tongue contact, and water uptake), whereas failed attempts are more heterogeneous and less visually well-defined. Interestingly, Qwen-3-VL-30B exhibited relatively better performance in disease-relevant conditions (early stroke: 0.8±0.16; later stroke: 0.73±0.17; not statistically significant), suggesting enhanced sensitivity to altered motor behaviors and increased utility in studies of neurological impairment. Although lower than expert human performance, this level of accuracy provides a scalable, automated baseline for high-throughput physiological studies—particularly those involving large-scale datasets—while maintaining sufficient statistical power and reducing manual labor.

By contrast, the smaller Qwen3-VL-8B and the VideoLLaMA3 (a 7B model) models constituted a significantly lower performance tier. Their failure was not merely a matter of lower accuracy but a profound classification bias, evident from their near-zero F1-scores for “success” trials (Fig. S3 in the Supplementary Material). These models effectively learned to default to a "failure" classification, rendering them practically useless for an analysis that requires the correct identification of both outcomes, even after. To avoid bias due to class imbalance, we subsampled the dataset to achieve equal representation of successful and failed trials during evaluation. The performance gap between the top and bottom models was statistically significant (p<0.001), suggesting that a certain level of model complexity and parameter size is necessary to capture and describe the visual details of complex motor behaviors.

Although these large video-language models demonstrate robust zero-shot capabilities, our analyses highlight critical limitations and structural requirements for their successful deployment in behavioral classification. To investigate the effects of the quality of input, we tested the reliance of Qwen3-VL-30B on specific visual information. Our findings reveal that the model’s accuracy is highly dependent on image quality, with performance significantly degrading as effective video resolution was reduced to 25% and 10% of the original through bilinear interpolation. This indicates the importance of fine-grained visual details rather than just gross movements for video LLMs. At the original resolution, the imaging scale was 0.14 mm per pixel. This suggests that the minimal features required for accurate classification become degraded once the effective resolution drops to between 0.28 and 0.57 mm per pixel corresponding to 50% and 25% effective resolution, respectively. Moreover, selective blurring experiments revealed that orofacial cues are the most critical feature for classification. Although obscuring either paw (so it became an unrecognizable blur) did not significantly impact performance, blurring the facial region led to a significant drop in accuracy. However, when the input was restricted to only the isolated orofacial region, model performance collapsed, with all trials classified as failures. This indicates that although orofacial cues are necessary, they are insufficient without additional spatial context. Together, these findings suggest that, unlike specialized CNN-based approaches that rely on localized features,^37^ the model integrates both local features and broader spatial context to identify the biologically relevant endpoint of the task—the successful delivery of the water droplet to the mouth—mirroring human expert evaluation.

In addition to spatial fidelity, our temporal resolution experiments identify a critical bottleneck regarding effective sampling frequency. Models like Gemini natively sample based on video playtime rather than frame count, real-time videos of fast motor behaviors are severely under-sampled. Consistent with this, we find that playback rates of 1 to 2 frames per second yield the highest accuracy, whereas higher playback speeds, including the original 54 fps recordings, result in progressively reduced performance. These results indicate that increasing playback speed compresses larger portions of behavior into each sampled frame, impairing classification. To preserve sufficient temporal detail without exceeding the model’s context constraints, inputs should therefore be aligned with the model’s sampling behavior (most model default 1 frame per 1 s video play time), ensuring that key kinematic features are adequately represented across sampled frames.

Most impressively, the ability of Gemini 2.5 Pro to generate descriptive text that segments behaviors temporally offers a transformative opportunity for accelerating research. Even though not all descriptions generated are correct, a substantial fraction are and can be curated or confirmed to rapidly generate large amounts of high-quality training data. This process addresses a key challenge in developing video understanding systems: the creation of extensive, accurately labeled datasets for fine-tuning LLMs. The outputs could also support the training or refinement of other neural network–based approaches, such as A-SOiD,^14^ serving as a powerful first pass or complementary tool to conventional labeling platforms like BORIS.^38^ The ability of Gemini to perform complex segmentation tasks so effectively, where other tested models failed, is suspected to be due to its greater number of model parameters and assumed complexity. Although the model remains proprietary without a supporting publication on the architecture, the observed capabilities suggest an exceptionally large-parameter count, likely exceeding 200 billion, which may facilitate advanced video understanding abilities.

Given the rising spatial-temporal understanding capabilities of recent video LLMs,^39^^,^^40^ we further tested specifically the ability of Gemini on estimating the volume of a water drop, an object of interest in our task. Judging the relative size of an object within a single static image was effective, as evidenced by the model’s robust detection of drop numbers and a significant positive linear correlation of predicted size with actual volumes in the water drop. Unfortunately, the model’s ability to precisely quantify temporal volumetric changes (e.g., water being sucked from a drop over a period of time) was more limited. Accurate detection of dynamic alterations across time appears to require robust visual alterations in the drop meaning that gradual changes may go un-noticed by a video LLM. This is supported by the observed linear decrease in error for predicted percentage removed as the true amount of water removed increased. Therefore, for applications requiring the tracking of evolving amounts, enhanced visual fidelity or more pronounced physical transformations are critical for the model to perform reliably. Furthermore, this highlights a key limitation in off-the-shelf deployment: model output is highly sensitive to the initial query. Optimizing prompt design is a necessary strategy to improve classification accuracy and reduce hallucinations. Future implementations should explore structuring prompts to explicitly guide the model’s attention—for example, by instructing it to evaluate distinct temporal windows, anchor its analysis to stable background landmarks, or specifically attend to slower volumetric changes.

Consistent with recent findings by Stoppa et al. (2025),^41^ few-shot learning improves the efficacy of video-LLM models for automated behavioral segmentation in a trend analysis of identified events. Although system instructions with zero-shot prompting allow the model to recognize general actions, augmenting the prompt history with visual examples that established explicit spatial anchors (i.e., snout positions relative to the lever and spout) enhanced event detection. Consequently, the few-shot approach yielded event detections more consistent with human-annotated results, improving the identification of both ballistic movements (e.g., lever presses) and visually ambiguous, continuous actions (e.g., reward consumption). These results highlight that providing explicit visual context within the prompt architecture is a critical prerequisite for leveraging multimodal LLMs to achieve robust and reliable behavioral tracking.

The presented approach can substantially lower the technical barrier for behavioral neuroscience by enabling automated trial outcome classification without the need for specialized imaging hardware or sensor systems. To ensure reliable performance, users should consider the temporal and spatial resolution of their data in relation to the specific constraints of the Video-LLM being used. Rather than relying on fixed capture rates, it is important to account for the model’s internal frame sampling/tokenization and ensure that the input retains the key kinematic features of the behavior. Based on our findings, we recommend maintaining an effective spatial resolution of ∼0.28  mm per pixel and preserving an unobstructed field of view. When combined with iterative prompt refinement and the selection of representative frames spanning the full behavioral sequence, this approach supports standardized and reproducible analyses across laboratories. Ultimately, this approach enables high-throughput pipelines that significantly reduce both manual annotation labor and human bias. As large video-capable language models continue to advance rapidly, they hold the potential to transform behavioral neuroscience in a manner analogous to the impact of convolutional neural networks on image analysis, accelerating discovery and allowing researchers to focus on interpretation rather than scoring.

Although tools such as DeepLabCut provide high-fidelity kinematic tracking,^4^^,^^7^^,^^9^ relying on them introduces a two-step bottleneck: researchers must perform significant manual labeling and tuning first to train the pose-estimation model, and again to build the secondary classifiers that identify semantic outcomes.^10^^,^^14^^,^^15^ Because video-LLMs offer direct, zero-shot semantic classification, we hypothesize that their utility may extend far beyond the head-fixed reaching paradigm evaluated here. Although our current validation is limited to this specific task, the strong performance of these general-purpose models suggests that their pre-training on massive datasets enables a degree of cross-domain transfer. This opens exciting possibilities for freely moving, unconstrained behaviors where hardware sensors are impractical and coordinate tracking becomes computationally complex. Future work should systematically evaluate whether these models can reliably score complex social interactions (e.g., distinguishing aggressive mounting from social play), quantify naturalistic behavioral sequences (e.g., nest building or maternal care), or conduct automated severity scoring in disease models (e.g., seizure phenotyping in the open field).

Realizing this broader potential, however, must be balanced against practical deployment limitations. Most high-performing models require significant computational resources and are not broadly accessible in typical biological laboratories. Furthermore, full training is impractical, demanding millions of video–text pairs and data-center GPUs with over 300 GB of VRAM.^42^ Fortunately, open-source models such as Qwen enable domain adaptation through LoRA, which updates small low-rank adapter layers while keeping most parameters frozen significantly reduce GPU memory requirements. However, thousands of high-quality video–text pairs are still required, which is a highly manual process.^2^^,^^43^^,^^44^ In disease-relevant contexts such as stroke or neurodegenerative models, fine-tuning model parameters is likely necessary to capture altered kinematics and behavioral variability. As previously mentioned, Gemini’s accessibility further supports this process by facilitating the generation of high-quality image and video–text pairs. Interestingly, the strong performance of general-purpose video LLMs suggests that training on large-scale human-action datasets may allow cross-domain transfer to rodent behaviors, potentially complemented by incidental exposure to animal footage during pretraining;^25^^,^^45^ however, systematic evaluation is needed to confirm this possibility. Ultimately, leveraging additional open-source models across these diverse behavioral assays will be critical for assessing generalizability and determining how broadly the capabilities of video-LLMs extend beyond the current task, particularly in disease-relevant conditions where increased behavioral variability may impact performance.

## Supplementary Material

10.1117/1.NPh.13.3.036601.s01

10.1117/1.NPh.13.3.036601.s1

## Data Availability

All codes for video LLM and statistical analysis can be found at the following GitHub repository: https://github.com/tf4ong/videollm. All data are available at the open science frame-work repository: https://osf.io/24euy. LLMs such as Claude and Gemini were used in the assistance of code generation for statistical testing and visualization. All data and relevant scripts are available at the following open science framework and GitHub repositories https://osf.io/24euy and https://github.com/tf4ong/videollm, respectively.
