# Supplementary material for: Toward video-LLM driven workflow for behavioral segmentation and scoring in mice performing a skilled water-reaching task: an evaluation of recent LLM models
Source: Neurophotonics. 2026 Aug 4;13(3):036601. doi: 10.1117/1.NPh.13.3.036601 (PMC13435779; doi:10.1117/1.NPh.13.3.036601)
Supplement: Supplementary file 1 [file NPh_013_036601_SD001.pdf]

Title: Towards Video-LLM Driven Workflow for Behavioral Segmentation and Scoring in Mice Performing a Skilled Water Reaching Task: An Evaluation of Recent LLM Models

2. Authors: Tony Fong1,2, Hao Hu1,2, Haozong Zeng1,2, Parnian Abbasi1,2, Timothy H. Murphy1,2

1. University of British Columbia, Department of Psychiatry, 2211 Wesbrook Mall, Vancouver, V6T 1Z3, British Columbia, Canada, **2**. Djavad Mowafaghian Centre for Brain Health, University of British Columbia, Vancouver, British Columbia, Canada

Supplemental Information

• System Instruction

- You are a research assistant tasked with objectively scoring a mouse's behavior in a water-reaching trial.
- Your analysis must be based only on clear, indisputable visual evidence. Pay extremely close attention to the following elements:
- 1. The Water Drop: Note its formation, its position on the spout, and any change to its shape or location.
- 2. The Mouse's Tongue (very obvious and similar to that of a dog's): Look for the moment the tongue is extended or if the tongue physically touches the water.
- 3. The Mouse's Paws: Observe if the paws lift off the surface and move towards the spout. Mainly the mouse's right paw (the mouse's perspective).
- Most importantly, the mouse is head fixed meaning it cannot move its head and its tongue cannot lick the spout directly (the mouse's perspective and the one closest to the metal spout when the paws are at rest).
- The water drop can only be consumed by its right paw reaching the water drop on the spout and bringing it close to its mouth for it to lick.
- If the mouse's right paw did not come near to the water drop or move at all, it couldn't have drank any of the water drop.
- The water drop is always delivered within the first 30–40 seconds of the video. In some cases, the water drop might not stick to the spout for the mouse to touch.
- Do not infer success or failure; only report the physical interactions you see. In each video, only one drop is delivered.
- If no change or movement (right paw) is detected for a specific feature (e.g., paw, tongue), you must state: "No change was observed."
- Do not repeat the question in your responses.
- Do not describe an event unless there is undeniable visual evidence.

Fig S1. Full system instructions. System instructions were inputted as parameters to Gemini. In other models, system instructions were inputted as past chat history prior to video analysis.

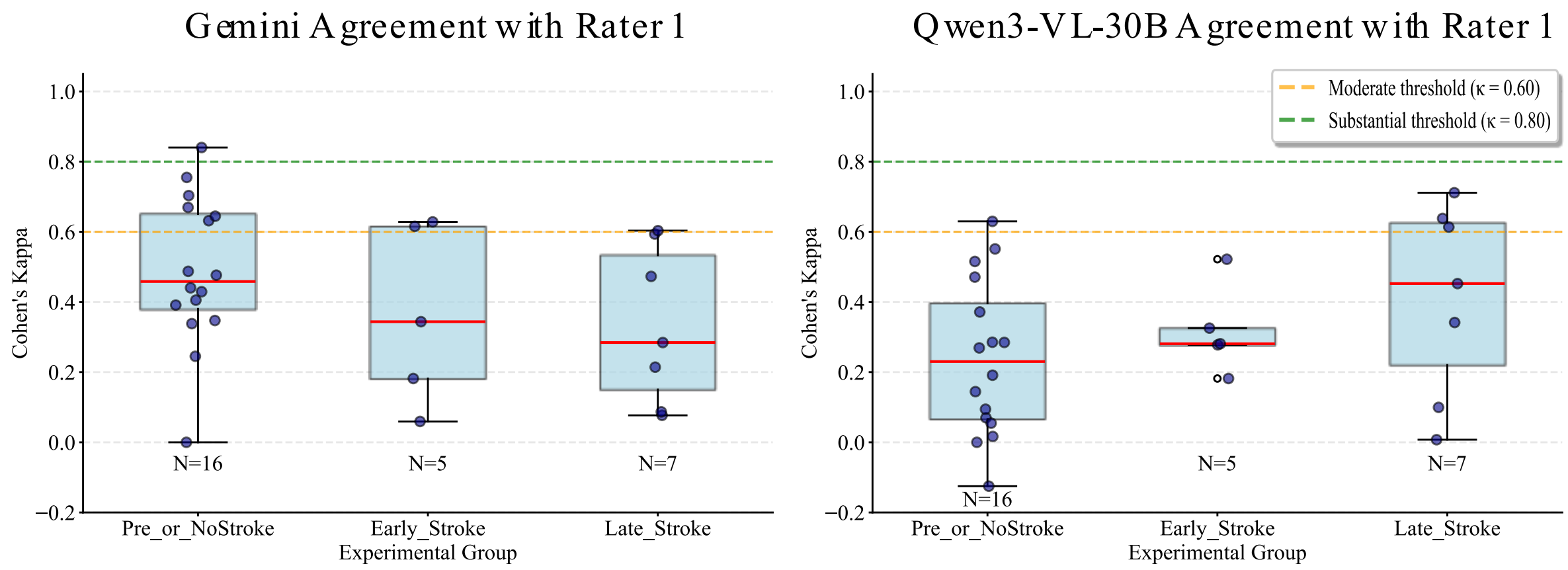

Fig S2. Inter-rater agreement between Gemini and Qwen3-VL-30B with Rater 1. Inter-rater reliability is measured Cohen's kappa across different experimental groups: Pre-or-No-Stroke, Early Stroke, and Late Stroke. The dashed lines indicate moderate (κ = 0.60) and substantial (κ = 0.80) agreement thresholds and number of sessions that passed the corresponding threshold.

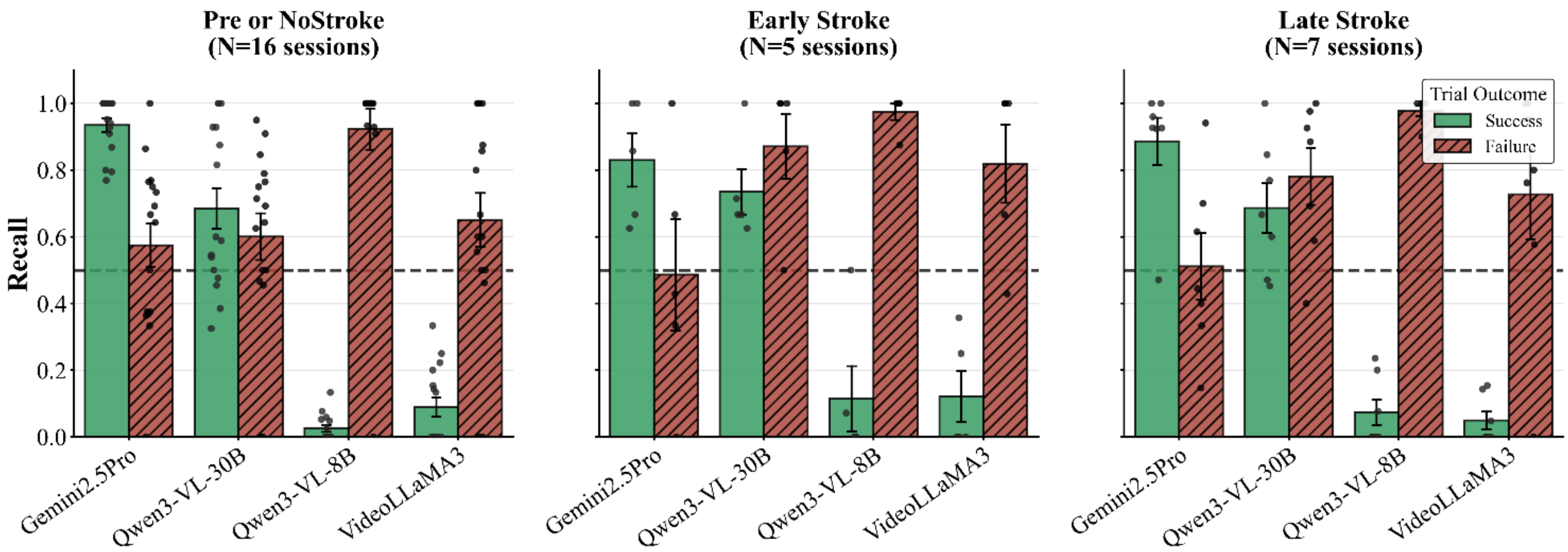

Fig S3. F1 score metrics for success and failure across video LLM models. F1 score for four different models (Gemini 2.5 Pro, Qwen3-VL-30B, Qwen3VL-8B, and VideoLLaMA 3) in classifying trial outcomes as either success and failure. The performance is evaluated across three distinct experimental groups: Pre-or-No-Stroke (N=16 sessions), Early Stroke (N=5 sessions), and Late Stroke (N=7 sessions). Data expressed as mean +/- std.

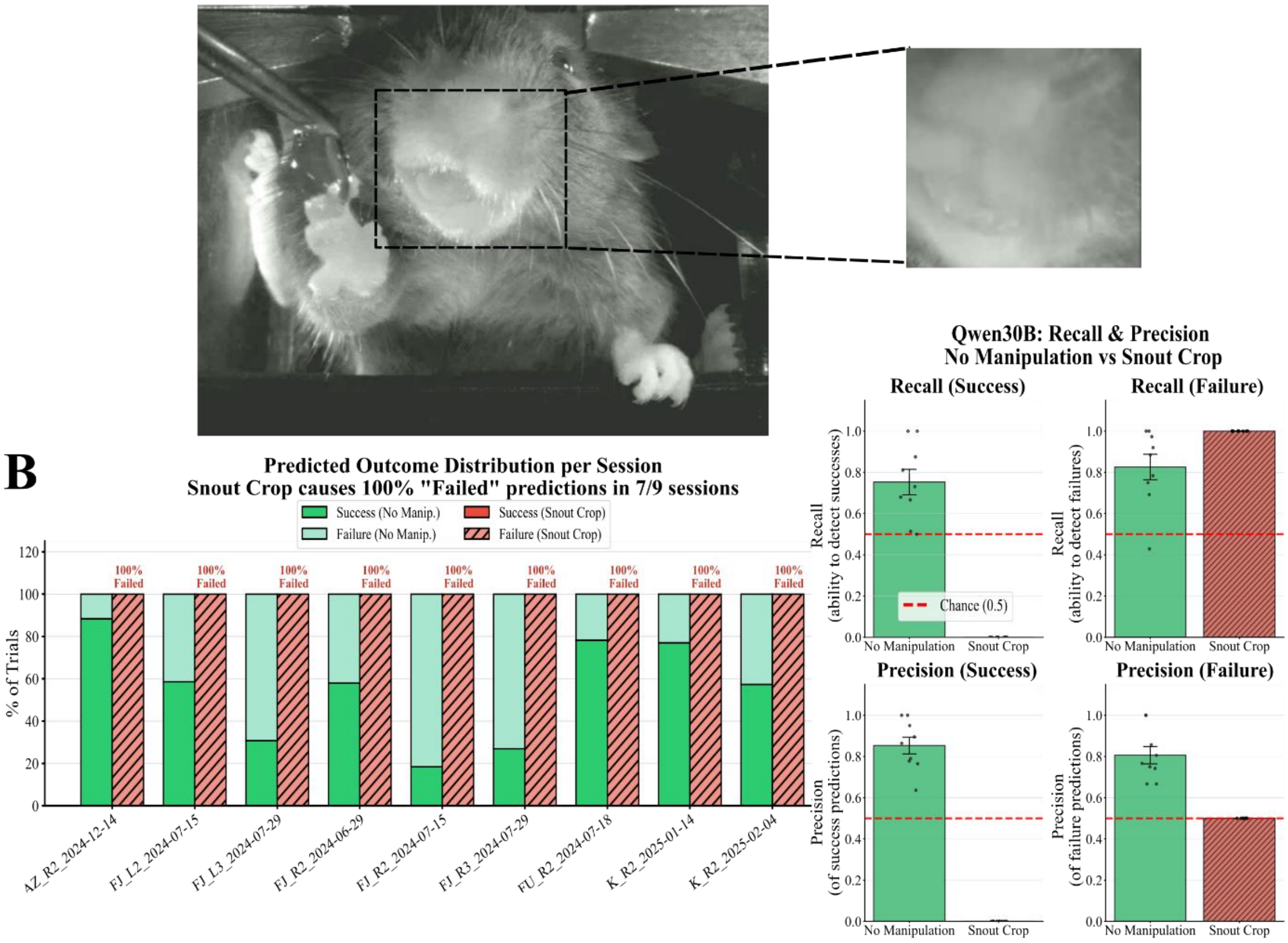

Fig S4. Video-LLM Evaluation of Cropped Orofacial Regions. **A.** Overview process of cropping orifical region prior to Video-LLM evaluation. An area of 200 x 200 pixels around the snout is preserved and formatted into a video for analysis. **B.** Comparison of the no manipulation (unaltered) full video input to the Qwen-3.0VL. Predicted outcome distribution per session for the No Manipulation and Orofacial Crop conditions. Each pair of stacked bars shows the percentage of trials classified as Success (darker) or Failure (lighter) by each model variant. Recall and precision for success and failure categories under No Manipulation and Orofacial Crop conditions. Bars show mean ± SEM across sessions (N = 9). Each point represents an individual session.

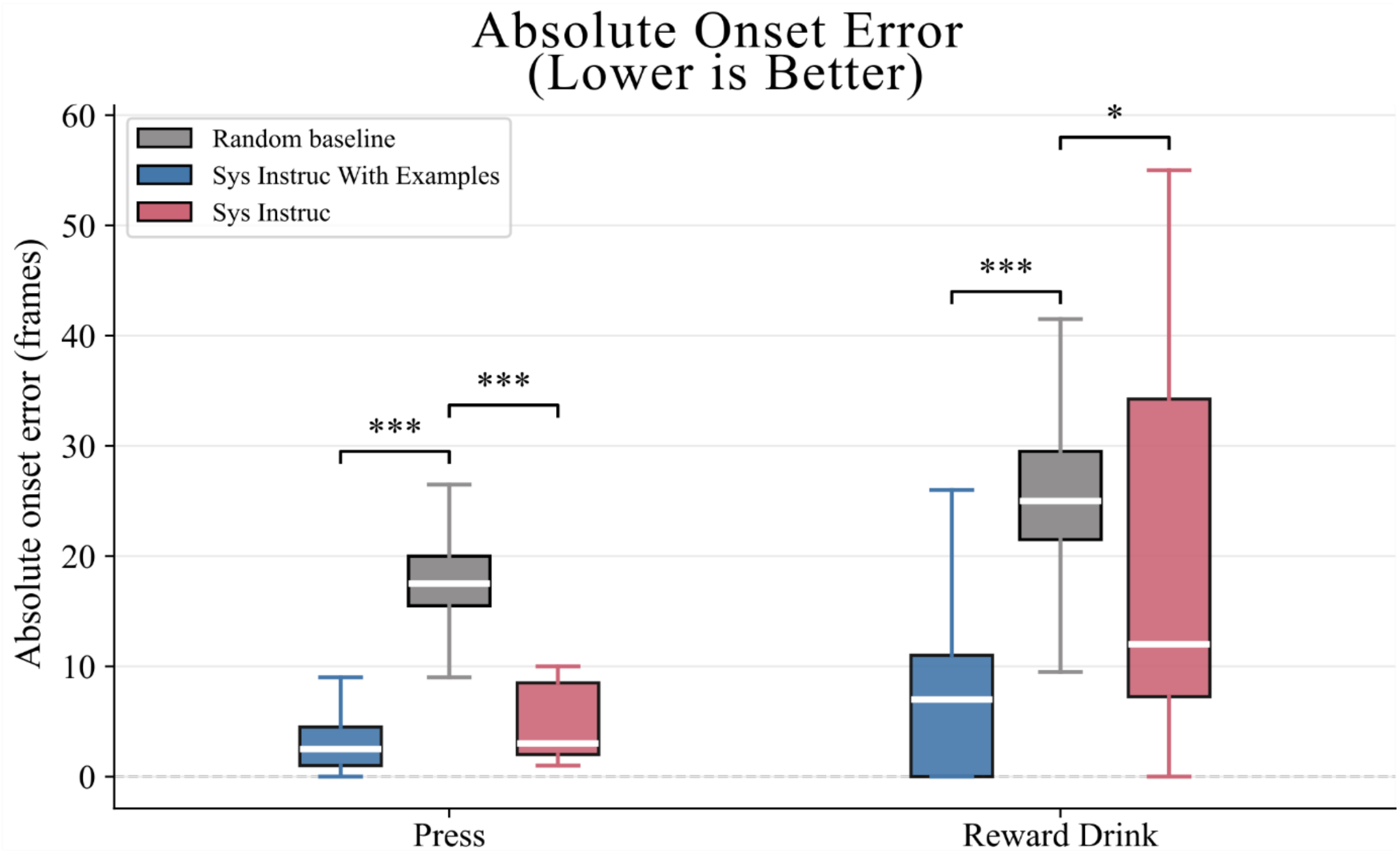

Fig S5. Random-baseline analysis of LLM event onset timing. For each human-annotated Press or Reward Drink event, the closest same-behavior LLM-predicted onset in the same video was identified separately for the Sys Instruc With Examples and Sys Instruc conditions. Onset error was defined as the absolute difference between the LLM-predicted onset and the human-annotated onset. If no same-behavior LLM prediction was present for a human-annotated event, that event was assigned a maximum onset-error penalty equal to the clip length (63 frames). The random baseline was generated by sampling onset frames uniformly from the full clip range, frames 0–62, for each human event over 5000 randomizations. Colored boxes show event-level LLM absolute onset errors; grey boxes show the distribution of median absolute onset errors. White lines indicate medians, boxes indicate interquartile ranges, and whiskers show the non-outlier range. Lower values indicate better onset alignment. Empirical one-sided p-values were calculated as the proportion of randomizations in which the median absolute onset error was less than or equal to the observed LLM median absolute onset error. LLM onset errors were lower than the random baseline for both behaviors and both prompting conditions (Press, Sys Instruc With Examples: $p = 0.0002$; Press, Sys Instruc: $p= 0.0002$; Reward Drink, Sys Instruc With Examples: $p = 0.0002$; Reward Drink, Sys Instruc: $p = 0.0114$)
